# Multiparametric MRI-based radiomics for preoperative prediction of parametrial invasion in early-stage cervical cancer

**DOI:** 10.3389/fonc.2025.1604749

**Published:** 2025-08-01

**Authors:** Chongshuang Yang, Man Li, Xin Yi, Lin Wang, Guangxian Kuang, Chunfang Zhang, Benyong Yao, Zhihong Qin, Tianliang Shi, Qiang Jiang

**Affiliations:** ^1^ Department of Radiology, Tongren People’s Hospital, Tongren, Guizhou, China; ^2^ Department of Research and Development, Shanghai United Imaging Intelligence Co., Ltd., Shanghai, China; ^3^ Department of Cardiology, Affiliated Hospital of North China University of Science and Technology, Tangshan, Hebei, China

**Keywords:** cervical cancer, magnetic resonance imaging, radiomics, parametrial invasion, multiparameters

## Abstract

**Objective:**

The aim of this study was to evaluate the performance of radiomics based on multiparametric magnetic resonance imaging (MRI) for the preoperative prediction of parametrial invasion (PMI) in cervical cancer (CC).

**Materials and methods:**

This retrospective study included 110 consecutive patients with International Federation of Obstetrics and Gynecology (FIGO) stage IB–IIA CC. Patients were randomly divided into a training and a testing cohort in an 8:2 ratio. The region of interest (ROI) was manually delineated. Radiomics features were extracted separately from T2-weighted imaging (T2WI), diffusion-weighted imaging (DWI), apparent diffusion coefficient (ADC), and contrast-enhanced T1-weighted imaging (T1C). Feature selection was performed using the correlation coefficient, recursive feature cancellation, and the least absolute shrinkage and selection operator algorithm. Radiomics models based on single-sequence, dual-sequence, and multi-sequence combinations were then constructed. Model performance was assessed using receiver operating characteristic (ROC) curve analysis. The DeLong test was used to compare the area under the curve (AUC), supplemented by net reclassification improvement and comprehensive discrimination improvement measures.

**Results:**

A total of 2,264 radiomics features were initially extracted. After feature selection, 7, 10, 6, and 8 valid features were retained from T1C, T2WI, ADC, and DWI sequence, respectively. A total of 15 radiomics models were developed, namely, 4 single-sequence models, 6 double-sequence models, and 5 multi-sequence models. All models showed good classification performance for PMI in both training and testing cohorts, with an AUC ranging from 0.755 to 1.000 in the training cohort and from 0.758 to 0.917 in the testing cohort. Among them, the T1C+ADC+DWI model demonstrated the best diagnostic performance, significantly outperforming all other models (*p* < 0.05), with the highest AUC in both training and testing cohorts (training: 1.000, testing: 0.917).

**Conclusion:**

Radiomics based on multiparametric MRI can effectively predict PMI status in patients with early-stage CC, offering valuable support for individualized treatment planning and clinical decision-making.

## 1 Introduction

Cervical cancer (CC) is the most common malignancy of the female reproductive system (1). Parametrial invasion (PMI) refers to the spread of cancer cells beyond the cervix into the surrounding parametrium. PMI is a key factor in the International Federation of Obstetrics and Gynecology (FIGO) staging system, which plays an important role in determining treatment strategies (2). Surgery is typically recommended for patients with CC without PMI (stage IIA and below), while concurrent chemoradiotherapy is the preferred approach for patients with PMI (stage IIB and above) (3). Therefore, accurate pre-treatment assessment of PMI is of great clinical significance for personalized treatment planning in CC.

Magnetic resonance imaging (MRI) is the preferred imaging modality for the diagnosis, staging, and treatment evaluation of CC, and it has been incorporated into the FIGO staging system as a recommended assessment tool (4). However, MRI-based evaluation of PMI by the radiologist has a relatively high false-positive rate, particularly in cases with large tumor or indistinct tumor boundaries caused by compression or inflammation (5, 6).

Radiomics combines image processing and big data analysis with medical imaging, enabling the large-scale extraction of quantifiable image features from lesions and the generation of imaging biomarkers that reflect subtle characteristics of tumors. This approach facilitates comprehensive analysis of the associations between imaging data and genetic, pathological, and clinical information (7). Radiomics has been applied to predict lympho-vascular space invasion (LVSI) (8), deep stromal invasion (9), and lymph node metastasis (10) in CC. Recently, several studies (11–13) have demonstrated that MRI-based radiomics offers significant advantages in predicting PMI in CC. However, to date, no studies have reported the use of multiparametric MRI-based radiomics for PMI prediction. Therefore, in this study, we aimed to develop radiomics models based on multiparametric MRI to predict PMI status preoperatively and support personalized treatment decision-making in patients with early-stage CC.

## 2 Materials and methods

This study was approved by the Ethics Committee of Tongren People’s Hospital on 23 May 2024, exempting the subjects from informed consent.

### 2.1 Patient collection

**Figure 1:**
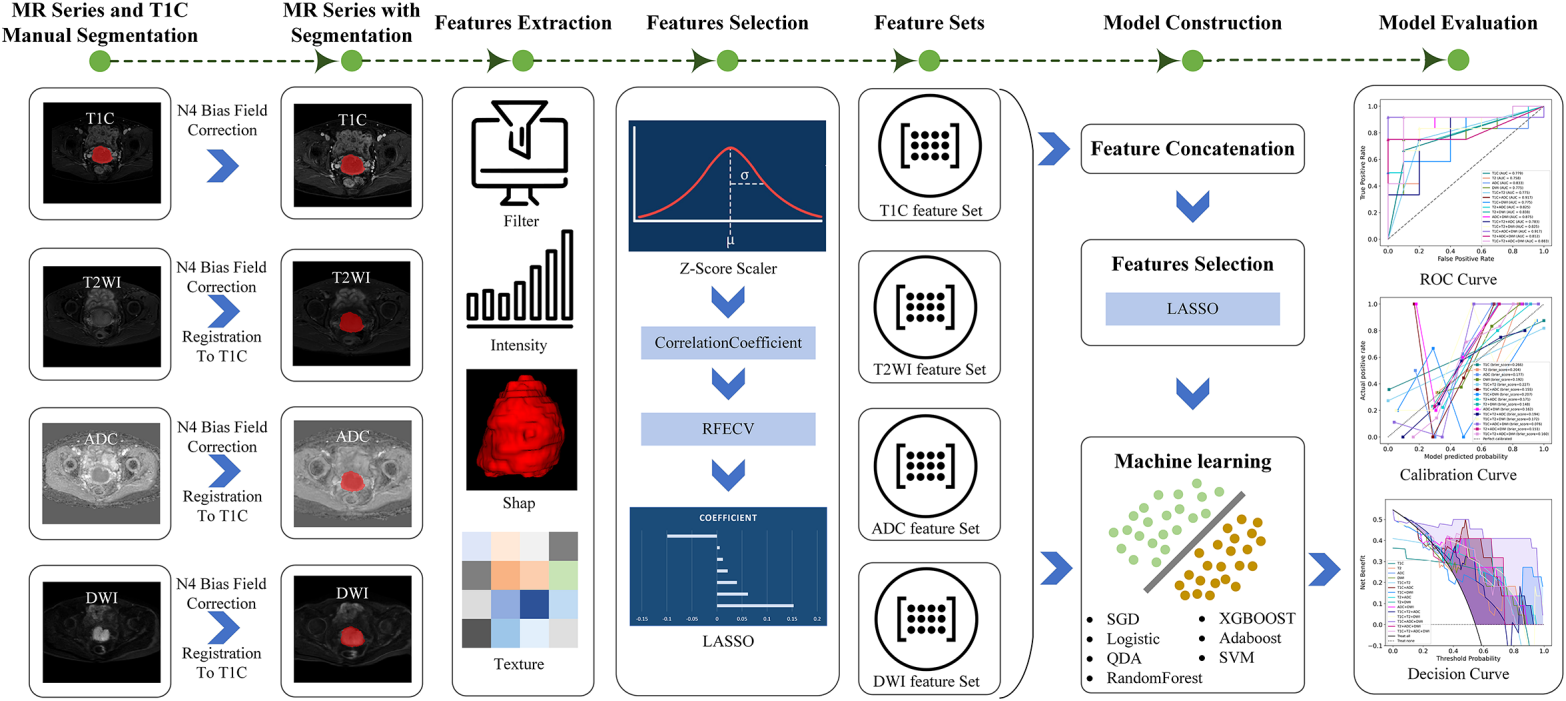
The overall workflow for this study.

This study retrospectively analyzed the preoperative MRI images and clinicopathological data of patients with CC with FIGO stage IB1–IIA who underwent radical resection in our hospital from March 2020 to October 2024. The inclusion criteria were as follows: (1) Patients were pathologically diagnosed with CC and had a clearly documented PMI status. (2) Patients had not received radiotherapy or chemotherapy before MRI scanning. (3) Patients underwent scanning with routine MRI, diffusion-weighted imaging (DWI), and contrast-enhanced T1-weighted imaging (T1C). The exclusion criteria were as follows: (1) MRI images that did not meet the requirements for tumor segmentation. (2) The time interval between MRI scanning and surgery was more than 2 weeks. The overall workflow for this study is shown in **Figure 1**.

### 2.2 Image acquisition

MRI scanning was performed on a Philips Ingenia 3.0 T MR machine. A total of four types of MRI images were used in this study, namely, axial T2-weighted imaging (T2WI), DWI, apparent diffusion coefficient (ADC), and T1C. The parameters of MRI scanning were as follows: T2WI: repetition time (TR)/echo time (TE) = 2,500/70 ms, field of view (FOV) = 23 × 36 cm, matrix = 264 × 320, slice thickness/spacing = 5/1 mm. T1WI: TR/TE = 485/8 ms, FOV = 32 × 28 cm, matrix = 300 × 377, slice thickness/spacing = 5/1 mm. DWI: TR/TE = 3,960/73 ms, FOV = 23 × 22 cm, matrix = 68 × 78, slice thickness/spacing = 3.3/0.33 mm, *b*-values were 0 and 800 s/mm^2^. After conventional pelvic MRI scanning, the patient was kept in the same position, and a contrast agent (0.1 mmol/kg) was injected through the elbow vein. Multislice dynamic contrast-enhanced images covering the lesion tissue were acquired, with delayed images obtained at 2 and 5 min post-injection. The scanning parameters for contrast-enhanced sequences were consistent with those of T1WI. After scanning, the ADC images were automatically reconstructed.

### 2.3 Manual segmentation and image preprocessing

#### 2.3.1 Manual segmentation

The regions of interest (ROIs) were manually segmented slice by slice on axial T1C images using ITK-SNAP software (University of Pennsylvania Image Computing and Science Laboratory, version 3.6.0). Two radiologists with over 5 years of work experience independently performed the delineation. Inter-observer agreement was assessed by calculating the Dice similarity coefficient, which yielded a value of 0.8566 ± 0.0442, demonstrating good consistency. For cases with low agreement (Dice < 0.7), the final ROI was reviewed and adjudicated by a senior radiologist with more than 15 years of experience to ensure segmentation accuracy and reproducibility.

#### 2.3.2 Image preprocessing

To eliminate intensity inhomogeneity commonly present in MR images, we first applied the N4 bias field correction method to all sequences. Subsequently, an adaptive normalization procedure was performed for each image to remove extreme voxel values (above the 99th percentile and below the 1st percentile), thereby minimizing the influence of outliers. Next, the intensity values were normalized to a range of 0 to 1 using min–max normalization to ensure data consistency and comparability across patients. For multi-sequence image analysis, the T1C sequence was used as the reference image, while T2WI, DWI, and ADC sequences were designated as moving images. These were spatially aligned to the T1C sequence using the symmetric normalization (SyN) algorithm, which integrates both affine and non-linear (deformable) transformations and employs mutual information as the optimization criterion. This registration process ensures spatial correspondence across sequences, laying a robust foundation for subsequent multi-parametric data fusion and analysis.

### 2.4 Radiomics feature extraction and feature selection

#### 2.4.1 Feature extraction

This study was conducted using the uAI Research Portal (United Imaging Intelligence, China, Version: 20240430), which is built on the Python programming language (version 3.7.3, https://www.python.org) and incorporates the widely used PyRadiomics package (https://pyradiomics.readthedocs.io/en/latest/index.html). Our study involved four sequences: T1C, T2WI, ADC, and DWI, and extracted 14 shape features, 18 first-order statistical features that describe the distribution of voxel intensities, and 72 texture features that characterize the spatial gray-level distribution of the pixel neighborhoods. To further expand the feature set, we applied 24 different filtering processes to the original images, including but not limited to mean filtering, Gaussian filtering, logarithmic filtering, and wavelet transformation. Subsequently, we extracted an additional 432 first-order statistical features and 1,728 texture features from these filtered images. In total, 2,264 radiomics features were extracted from each sequence.

#### 2.4.2 Feature selection

Feature selection involves identifying the most discriminative features from the original feature set to reduce dimensionality. For the 2,264 radiomics features, *z*-score normalization was applied to normalize the features. Subsequently, the correlation coefficient between each feature and the presence or absence of PMI was calculated, retaining only those with a significance level of *p* < 0.05. Following this, a recursive feature elimination method was employed to iteratively remove features. This method employed a support vector machine (SVM) as the base estimator and iteratively removed redundant features. In each iteration, the model performance of the feature subset was evaluated via 5-fold cross-validation until the number of remaining features reached the predefined threshold. Finally, to further optimize feature selection, the least absolute shrinkage and selection operator (LASSO) was applied to identify the subset of features most strongly associated with the existence of PMI.

### 2.5 Classification model building

Based on the radiomics features selected from four sequences (T1C, T2WI, ADC, and DWI), four single-sequence models were constructed. By combining radiomics features from two to four sequences and performing feature selection again using LASSO, six dual-sequence models, four triple-sequence models, and one four-sequence model were subsequently developed. In total, 15 models were constructed. For each model, we evaluated the area under the curve (AUC), sensitivity, specificity, accuracy, precision, and F1 score. We also generated receiver operating characteristic (ROC) curves, calibration curves, and decision curves. Ultimately, we selected the best-performing model for predicting PMI in CC by comparing the diagnostic performance of all models.

### 2.6 Statistical analyses

The data were imported into the statistical analysis module of the uAI Research Portal software for correlation analysis, in which the differences in continuous variables and quantitative data in patients with positive and negative PMI were compared using either the *t*-test or the Mann–Whitney *U* test, depending on the data distribution. Categorical variables and the incidence of PMI were compared using the chi-square test or Fisher’s exact test. A *p*-value < 0.05 was considered statistically significant. The diagnostic performance of the models was evaluated using ROC analysis. The DeLong test was used to compare AUC, and the analysis was supplemented by net reclassification improvement (NRI) and integrated discrimination improvement (IDI) measures.

## 3 Results

### 3.1 Characteristics of patients

A total of 110 patients aged 38–68 years were included in this study, with a median age of 52 years. Among the 110 enrolled patients, 60 were positive for PMI and 50 were negative. According to the ratio of 8:2 (random seed = 20), patients were randomly assigned to the training cohort (*n* = 88) and the testing cohort (*n* = 22). The clinical characteristics of the patients are shown in **Table 1**. In the training cohort, a significant difference was observed in LVSI status between the PMI-positive and PMI-negative groups (*p* < 0.001). There were no significant differences in age, tumor size, pathological type, and degree of differentiation between the two groups (*p* > 0.05).

**Table 1:** The general clinical information of patients.

Characteristics		Training cohort (*n* = 88)			Testing cohort (*n* = 22)		
		PMI-negative (*n* = 40)	PMI-positive (*n* = 48)	*p*	PMI-negative (*n* = 10)	PMI-positive (*n* = 12)	*p*
Age		54.05 ± 10.04	56.52 ± 9.71	0.245	57.80 ± 8.97	52.92 ± 10.03	0.247
Tumor size (mm)		31.75 ± 12.88	32.44 ± 13.95	0.812	30.60 ± 6.33	33.00 ± 11.60	0.566
**Type (%)**				0.777			0.6766
	SCC	35 (87.50)	41 (85.417)		8 (80.00)	8 (75.00)	
	AC	5 (12.50)	7 (14.583)		2 (20.00)	4 (25.00)	
Degree of differentiation (%)				0.676			0.576
	High	8 (20.00)	7 (14.583)		1 (10.000)	3 (25.00)	
	Middle	26 (65.00)	31 (68.750)		6 (60.000)	7 (58.33)	
	Low	6 (15.00)	10 (16.67)		3 (30.000)	2 (16.67)	
LVSI (%)				<0.001			0.198
	Positive	36 (90.00)	21 (43.75)		7 (70.00)	4 (25.00)	
	Negative	4 (10.00)	27 (56.25)		3 (30.00)	8(75.00)	
FIGO staging				<0.505			<0.852
	I	15 (37.50)	23 (47.92)		3 (30.00)	5 (41.67)	
	IA	19 (47.50)	17 (35.41)		6 (60.00)	6 (50.00)	
	IIB	6 (15.00)	8 (16.67)		1 (10.00)	1 (8.33)	

AC, adenocarcinoma; FIGO, International Federation of Gynecology and Obstetrics; PMI, parametrial invasion; SCC, squamous cell carcinoma; LVSI, lymphovascular space invasion.

### 3.2 Single modality features selection

**Figure 2:**
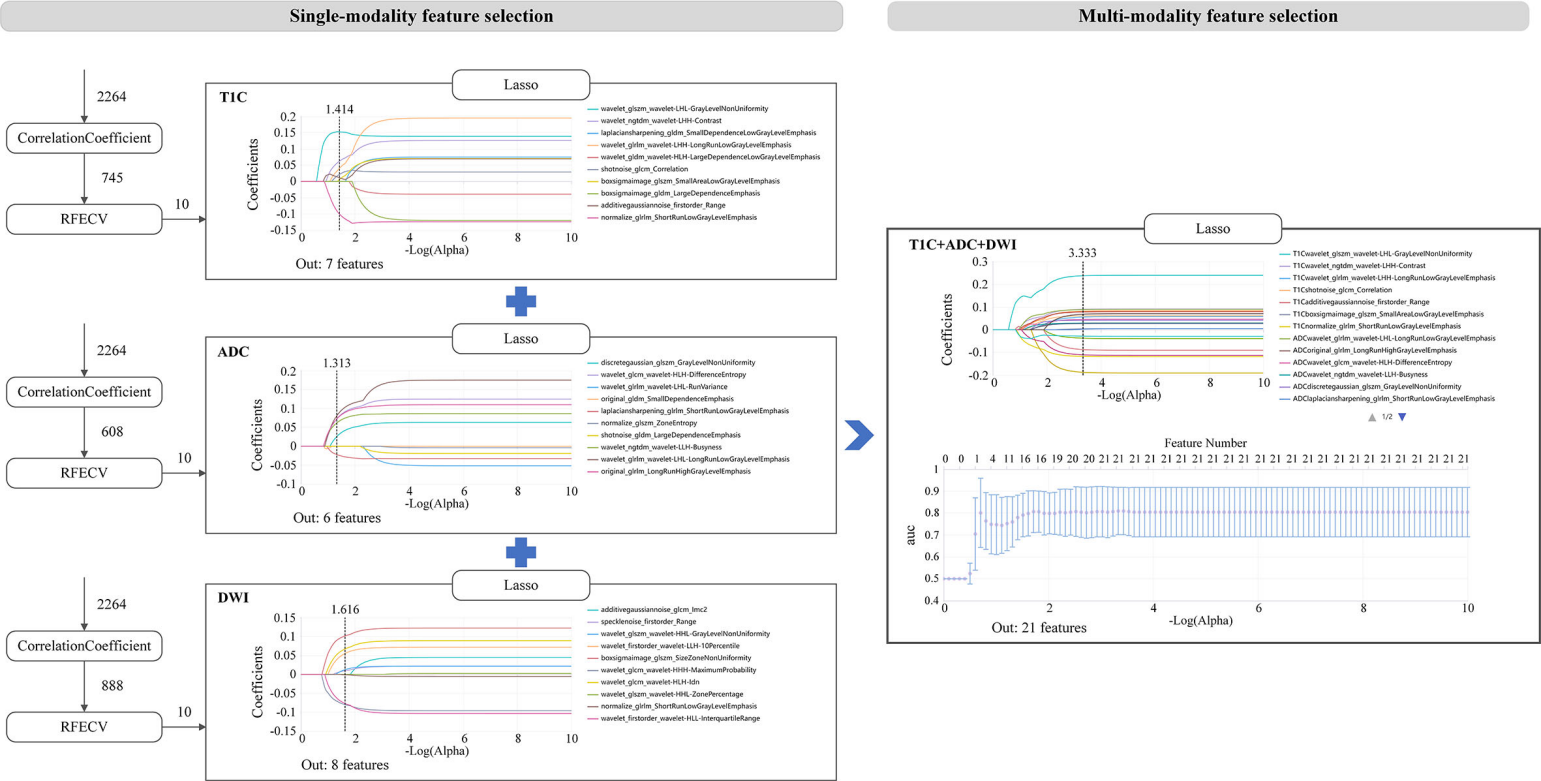
Radiomics features screened from single-modality and multi-modality.

A total of 2,264 radiomics features were extracted from the ROIs of each sequence. Then, the correlation coefficient (*p* < 0.05), recursive feature elimination, and LASSO methods were used sequentially to screen the features (as shown in **Figure 2**). In the recursive feature elimination process, we selected the top 10 features for further analysis. In the end, 7, 10, 6, and 8 valid features were selected from T1C, T2WI, ADC, and DWI, respectively.

### 3.3 Multi-modality feature selection

**Figure 3:**
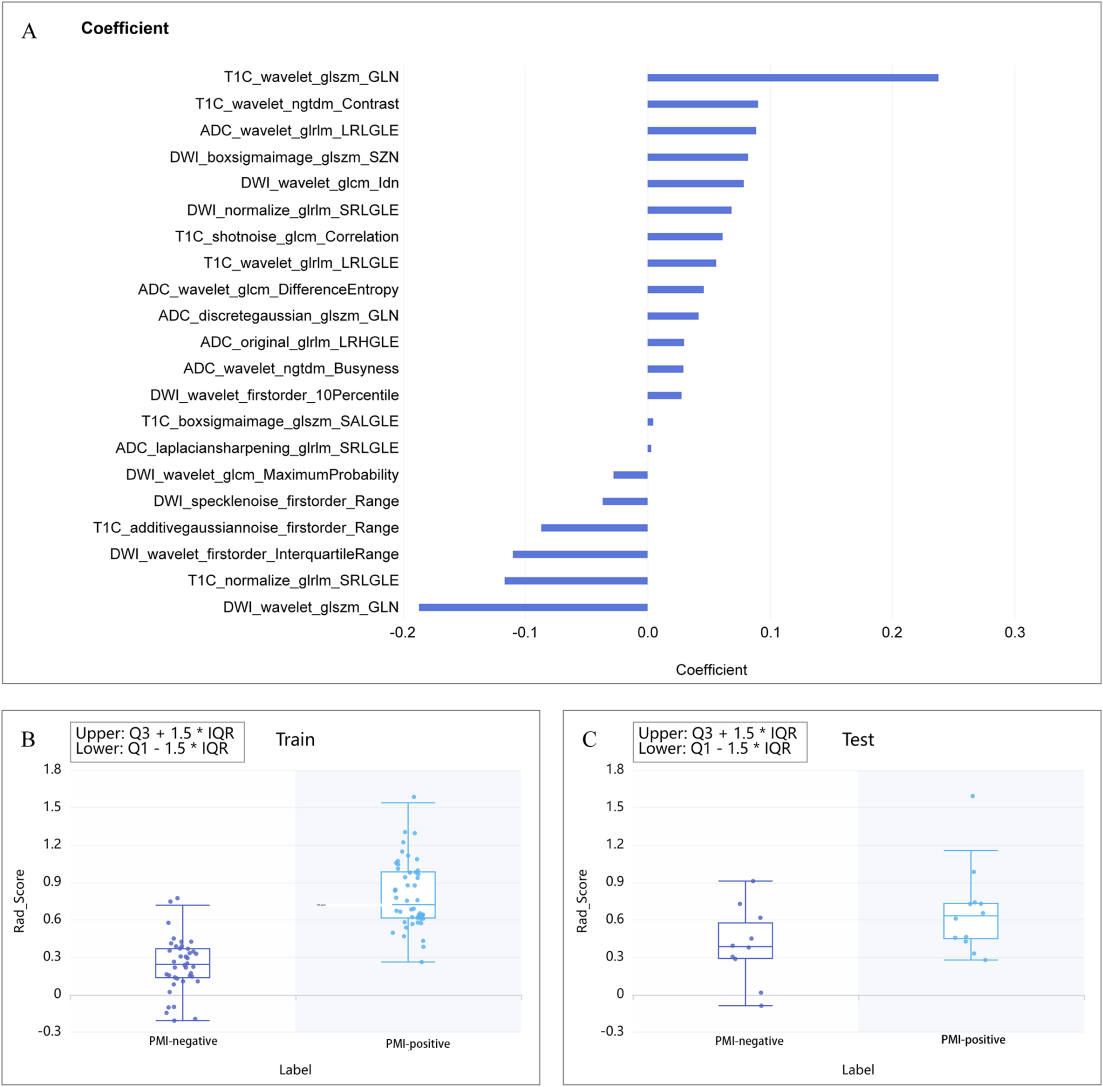
**(A)** Combination of selected radiomic features. **(B, C)** show the Rad-score distribution for the training and testing cohorts, respectively.

In the process of multi-sequence joint modeling, features from multiple sequences are concatenated, and feature selection is performed again using the LASSO method. Taking the T1C+ADC+DWI model as an example, 21 key features were retained after LASSO screening analysis, as shown in **Figures 2**, **3A**. These features include the first-order statistics, grayscale co-occurrence matrix (GLCM), grayscale run length matrix (GLRLM), gray-level size zone matrix (GLSZM), and neighboring gray-tone difference matrix (NGTDM). The Rad-scores of the screened features in the training and testing cohorts are shown in **Figures 3B, C**, respectively.

### 3.4 Performance of radiomics models

**Figure 4:**
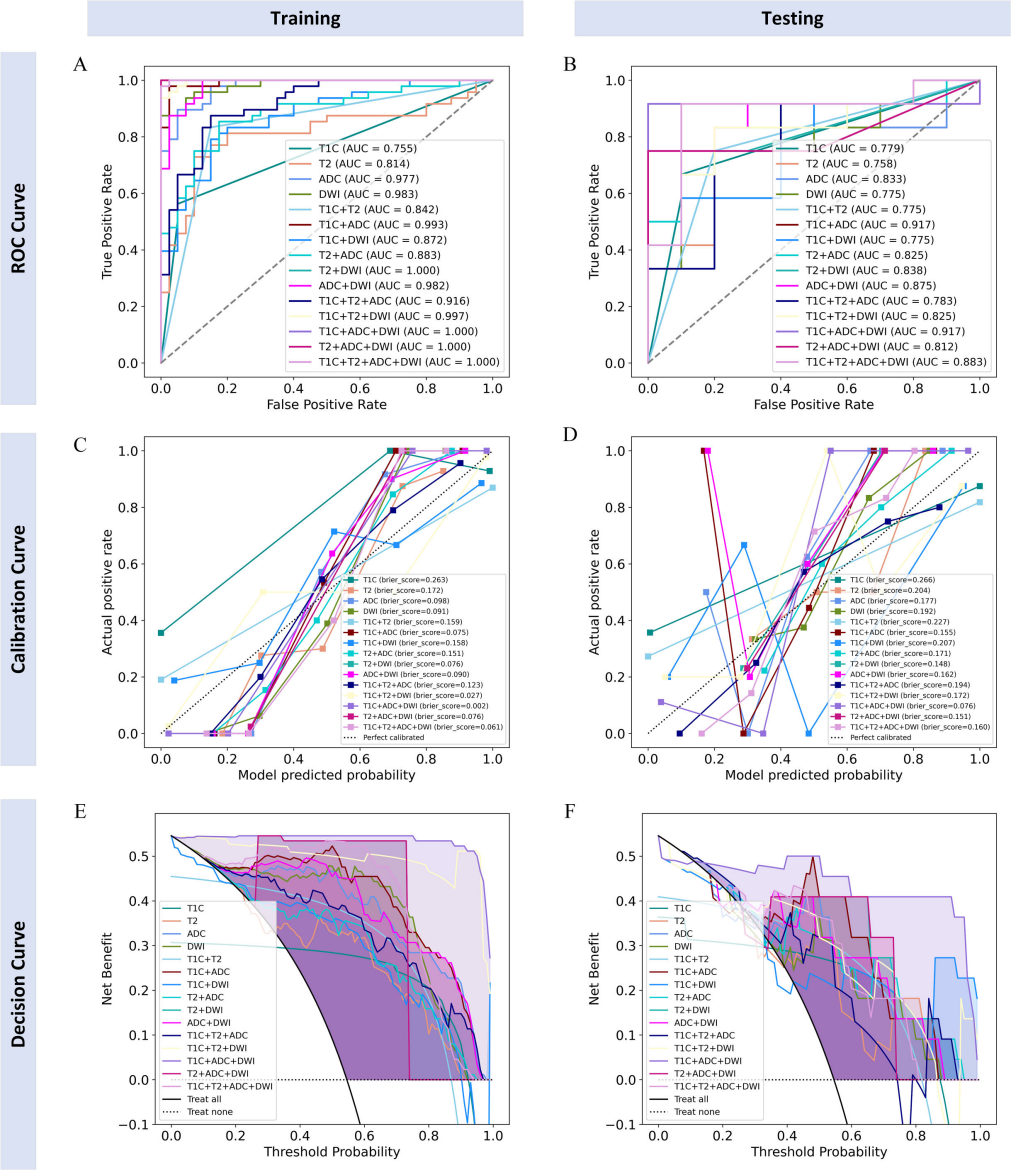
ROC curves, calibration curves, and decision curves of each radiomics model in training and testing cohorts. **(A, B)** ROC curves for the training and testing cohorts; **(C, D)** calibration curves for the training and testing cohorts; **(E, F)** decision curves for the training and testing cohorts.

The diagnostic performance of each radiomics model is shown in **Table 2**. All models demonstrated good classification performance for distinguishing positive and negative PMI in both the training and testing cohorts, with AUCs ranging from 0.755 to 1.000 in the training cohort and from 0.758 to 0.917 in the testing cohort. Among the four single-sequence models, the ADC model achieved the highest AUC in both the training and testing cohorts (training: 0.977; testing: 0.833). Among the six dual-sequence combination models, the T1C+ADC model had the highest AUC (training: 0.993; testing: 0.917). Among the combined models with three or more sequences, the T1C+ADC+DWI model demonstrated the strongest diagnostic performance, with an AUC of 1.000 in the training cohort and 0.917 in the testing cohort. Therefore, the T1C+ADC+DWI model was identified as the optimal model (**Figures 4A, B**).

**Table 2:** Performance of the radiomics models in the training and testing cohorts.

Model	Method	AUC	Sensitivity	Specificity	Accuracy	Precision	F1 score
Training cohort (*n* = 88)							
T1C	YeoJohnson_transformer >> SGD	0.755 (0.675–0.834)	0.562	0.950	0.739	0.931	0.701
T2	Quantile_transformer >> Logistic	0.814 (0.719–0.909)	0.771	0.800	0.784	0.822	0.796
ADC	L2_normalization >> Random Forest	0.977 (0.954–1.000)	0.896	0.950	0.920	0.956	0.925
DWI	L1_normalization >> Random Forest	0.983 (0.964–1.000)	0.958	0.900	0.932	0.920	0.939
T1C+T2	BoxCox_transformer >> SGD	0.842 (0.764–0.919)	0.833	0.850	0.841	0.870	0.851
T1C+ADC	L2_normalization >> Random Forest	0.993 (0.982–1.000)	0.979	0.975	0.977	0.979	0.979
T1C+DWI	Z_score_scaler >> QDA	0.872 (0.799–0.944)	0.792	0.825	0.807	0.844	0.817
T2+ADC	Min_max_scaler >> Logistic	0.883 (0.812–0.953)	0.812	0.825	0.818	0.848	0.830
T2+DWI	Z_score_scaler >> AdaBoost	1.000 (1.000–1.000)	0.979	1.000	0.989	1.000	0.989
ADC+DWI	L2_normalization >> Random Forest	0.982 (0.962–1.000)	0.917	0.900	0.909	0.917	0.917
T1C+T2+ADC	Quantile_transformer >> Logistic	0.916 (0.858–0.973)	0.833	0.850	0.841	0.870	0.851
T1C+T2+DWI	Z_score_scaler >> SVM	0.997 (0.992–1.000)	0.958	0.950	0.955	0.958	0.958
T1C+ADC+DWI	L2_normalization >> XGBOOST	1.000 (1.000–1.000)	1.000	1.000	1.000	1.000	1.000
T2+ADC+DWI	Z_score_scaler >> AdaBoost	1.000 (1.000–1.000)	0.979	1.000	0.989	1.000	0.989
T1C+T2+ADC+DWI	Z_score_scaler >> Gaussian Process	1.000 (0.998–1.000)	1.000	0.975	0.989	0.980	0.990
Testing cohort (*n* = 22)							
T1C	YeoJohnson_transformer >> SGD	0.779 (0.605–0.953)	0.583	0.900	0.727	0.875	0.700
T2	Quantile_transformer >> Logistic	0.758 (0.535–0.981)	0.833	0.700	0.773	0.769	0.800
ADC	L2_normalization >> Random Forest	0.833 (0.632–1.000)	0.750	1.000	0.864	1.000	0.857
DWI	L1_normalization >> Random Forest	0.775 (0.561–0.989)	0.750	0.900	0.818	0.900	0.818
T1C+T2	BoxCox_transformer >> SGD	0.775 (0.592–0.958)	0.750	0.800	0.773	0.818	0.783
T1C+ADC	L2_normalization >> Random Forest	0.917 (0.753–1.000)	0.750	1.000	0.864	1.000	0.857
T1C+DWI	Z_score_scaler >> QDA	0.775 (0.572–0.978)	0.583	0.800	0.682	0.778	0.667
T2+ADC	Min_max_scaler >> Logistic	0.825 (0.640–1.000)	0.750	0.800	0.773	0.818	0.783
T2+DWI	Z_score_scaler >> AdaBoost	0.838 (0.667–1.000)	0.750	1.000	0.864	1.000	0.857
ADC+DWI	L2_normalization >> Random Forest	0.875 (0.704–1.000)	0.583	1.000	0.773	1.000	0.737
T1C+T2+ADC	Quantile_transformer >> Logistic	0.783 (0.579–0.988)	0.750	0.800	0.773	0.818	0.783
T1C+T2+DWI	Z_score_scaler >> SVM	0.825 (0.643–1.000)	0.833	0.800	0.818	0.833	0.833
T1C+ADC+DWI	L2_normalization >> XGBOOST	0.917 (0.753–1.000)	0.917	1.000	0.955	1.000	0.957
T2+ADC+DWI	Z_score_scaler >> AdaBoost	0.812 (0.616–1.000)	0.750	1.000	0.864	1.000	0.857
T1C+T2+ADC+DWI	Z_score_scaler >> Gaussian Process	0.883 (0.721–1.000)	0.750	0.900	0.818	0.900	0.818

AUC, area under the curve; ADC, apparent diffusion coefficient; DWI, diffusion-weighted imaging; T2WI, T2-weighted imaging; T1C, contrast-enhanced T1-weighted imaging.

Next, we compared the diagnostic capability of the T1C+ADC+DWI model with the other 14 models (as shown in **Table 3**). In the training cohort, the diagnostic capability of the T1C+ADC+DWI model was significantly different from all other models (integrated NRI score > 0 and either NRI *p*-value < 0.05 or IDI *p*-value < 0.05). In the testing cohort, the diagnostic performance of the T1C+ADC+DWI model also differed significantly from all models (integrated NRI score > 0 and either NRI *p*-value < 0.05 or IDI *p*-value < 0.05) except for the T1C+T2 model (NRI *p*-value = 0.0717; IDI *p*-value = 0.4114).

**Table 3:** Performance comparison between the T1C+ADC+DWI model and all other models in both the training and testing cohorts.

Group	Old model	New model	AUC *p*-value	NRI score	NRI *p*-value	IDI score	IDI *p*-value
Training cohort	T1C	T1C+ADC+DWI	0	0.4875	0	0.449	0
	T2	T1C+ADC+DWI	0.0001	0.4292	0	0.6985	0
	ADC	T1C+ADC+DWI	0.0484	0.1542	0.0059	0.4951	0
	DWI	T1C+ADC+DWI	0.0759	0.1417	0.0107	0.4888	0
	T1C+T2	T1C+ADC+DWI	0.0001	0.3167	<0.001	0.2664	0.0007
	T1C+ADC	T1C+ADC+DWI	0.2008	0.0458	0.1541	0.4249	0
	T1C+DWI	T1C+ADC+DWI	0.0005	0.3833	0	0.4189	0
	T2+ADC	T1C+ADC+DWI	0.0012	0.3625	<0.01	0.6596	0
	T2+DWI	T1C+ADC+DWI	1	0.0208	0.3122	0.4957	0
	ADC+DWI	T1C+ADC+DWI	0.0877	0.1833	0.0031	0.467	0
	T1C+T2+ADC	T1C+ADC+DWI	0.0038	0.3167	<0.001	0.5436	0
	T1C+T2+DWI	T1C+ADC+DWI	0.2479	0.0917	0.0414	0.0623	0.0723
	T2+ADC+DWI	T1C+ADC+DWI	1	0.0208	0.3122	0.4957	0
	T1C+T2+ADC+DWI	T1C+ADC+DWI	0.4795	0.025	0.3112	0.4034	0
Testing cohort	T1C	T1C+ADC+DWI	0.2974	0.4333	0.0332	0.2221	0.2724
	T2	T1C+ADC+DWI	0.1118	0.3833	0.0205	0.553	0
	ADC	T1C+ADC+DWI	0.5555	0.1667	0.2963	0.4461	<0.01
	DWI	T1C+ADC+DWI	0.0927	0.2667	0.0630	0.5338	0
	T1C+T2	T1C+ADC+DWI	0.2848	0.3667	0.0717	0.1621	0.4114
	T1C+ADC	T1C+ADC+DWI	1	0.1667	0.1213	0.4404	0
	T1C+DWI	T1C+ADC+DWI	0.2472	0.5333	0.0041	0.3546	0.0123
	T2+ADC	T1C+ADC+DWI	0.1891	0.3667	0.0272	0.4702	0
	T2+DWI	T1C+ADC+DWI	0.3483	0.1667	0.1213	0.4083	0
	ADC+DWI	T1C+ADC+DWI	0.7455	0.3333	0.0143	0.433	<0.001
	T1C+T2+ADC	T1C+ADC+DWI	0.277	0.3667	0.0272	0.4757	0
	T1C+T2+DWI	T1C+ADC+DWI	0.2406	0.2833	0.0581	0.2888	0.0081
	T2+ADC+DWI	T1C+ADC+DWI	0.257	0.1667	0.1213	0.4173	0
	T1C+T2+ADC+DWI	T1C+ADC+DWI	0.5712	0.2667	0.0630	0.4578	0

AUC, area under the curve; ADC, apparent diffusion coefficient; DWI, diffusion-weighted imaging; T2WI, T2-weighted imaging; T1C, contrast-enhanced T1-weighted imaging; NRI, net reclassification improvement; IDI, integrated discrimination improvement.

Then, we continued to compare the T1C+T2 model with all other models (as shown in **Table 4**). In the training cohort, the diagnostic performance of the T1C+T2 model was not higher than that of most models (NRI score ≤ 0) except for the T1C, T2, T1C+DWI, and T2+ADC models (integrated NRI score > 0 and either NRI *p*-values < 0.05 or IDI *p*-values < 0.05). Therefore, the T1C+T2 model could not be considered an optimal model.

**Table 4:** Performance comparison between the T1C+T2 model and all other models in both the training and testing cohorts.

Group	Old model	New model	AUC *p*-value	NRI score	NRI *p*-value	IDI score	IDI *p*-value
Training cohort	T1C	T1C+T2	0.0309	0.1708	0.0322	0.1826	0.0228
	T2	T1C+T2	0.4753	0.1125	0.0805	0.4321	0
	ADC	T1C+T2	0.0003	−0.1625	0.0511	0.2286	0.0031
	DWI	T1C+T2	0.0002	−0.175	0.0294	0.2224	0.0019
	T1C+ADC	T1C+T2	0.0001	−0.2708	0.0002	0.1584	0.0344
	T1C+DWI	T1C+T2	0.3844	0.0667	0.4099	0.1524	0.0234
	T2+ADC	T1C+T2	0.2065	0.0458	0.584	0.3931	0
	T2+DWI	T1C+T2	0.0001	−0.2958	<0.001	0.2292	0.0031
	ADC+DWI	T1C+T2	0.0002	−0.1333	0.1332	0.2005	0.0083
	T1C+T2+ADC	T1C+T2	0.007	0	1	0.2772	0
	T1C+T2+DWI	T1C+T2	0	−0.225	0.0008	−0.2041	0.0020
	T1C+ADC+DWI	T1C+T2	0.0001	−0.3167	<0.001	−0.2664	0.0007
	T2+ADC+DWI	T1C+T2	0.0001	−0.2958	0.0003	0.2292	0.0040
	T1C+T2+ADC+DWI	T1C+T2	0.0001	−0.2917	<0.001	0.1369	0.0480
Testing cohort	T1C	T1C+T2	0.963	0.0667	0.7195	0.0599	0.7547
	T2	T1C+T2	0.8695	0.0167	0.9224	0.3909	0.0147
	ADC	T1C+T2	0.5256	−0.2	0.2473	0.2839	0.0745
	DWI	T1C+T2	1	−0.1	0.6747	0.3717	0.0494
	T1C+ADC	T1C+T2	0.1559	−0.2	0.2473	0.2782	0.0857
	T1C+DWI	T1C+T2	1	0.1667	0.4344	0.1925	0.2714
	T2+ADC	T1C+T2	0.6199	0	1	0.308	0.0570
	T2+DWI	T1C+T2	0.6537	−0.2	0.4049	0.2461	0.2329
	ADC+DWI	T1C+T2	0.4593	−0.0333	0.8873	0.2709	0.1543
	T1C+T2+ADC	T1C+T2	0.8939	0	1	0.3136	0.0169
	T1C+T2+DWI	T1C+T2	0.6708	−0.0833	0.7202	0.1267	0.5127
	T1C+ADC+DWI	T1C+T2	0.2848	−0.3667	0.0717	−0.1621	0.4114
	T2+ADC+DWI	T1C+T2	0.8047	−0.2	0.4049	0.2552	0.2190
	T1C+T2+ADC+DWI	T1C+T2	0.3048	−0.1	0.6021	0.2957	0.0708

AUC, area under the curve; ADC, apparent diffusion coefficient; DWI, diffusion-weighted imaging; T2WI, T2-weighted imaging; T1C, contrast-enhanced T1-weighted imaging; NRI, net reclassification improvement; IDI, integrated discrimination improvement.

Finally, we believe that the radiomics model based on the T1C+ADC+DWI sequences is the best model. The calibration curves of the radiomics model based on the T1C+ADC+DWI sequences showed good agreement over a wide probability range between the training and testing cohorts (**Figures 4C, D**). The decision curves show that the radiomics model based on the T1C+ADC+DWI sequences provides a greater net benefit in both the training and testing cohorts (**Figures 4E, F**).

## 4 Discussion

PMI is widely recognized as a risk factor for CC and has a direct impact on the prognosis of patients (14). Therefore, accurate preoperative evaluation of PMI is crucial. Although the disruption of the low-intensity cervical interstitial ring on T2WI can serve as a radiological indicator of PMI, the assessment largely depends on the radiologist’s experience and involves subjective interpretation. By contrast, radiomics can quantitatively extract high-dimensional features that reflect underlying biological information related to genes, proteins, and the tumor microenvironment, which are invisible to the naked eye (15). Compared with traditional visual assessment, radiomics offers a more objective and detailed evaluation of tumor pathology. In the present study, we retrospectively collected preoperative T2WI, ADC, DWI, and T1C images from patients with CC. Radiomics features were extracted and screened from each imaging modality, and predictive models were constructed based on single-sequence, double-sequence, and multi-sequence combinations. The diagnostic performance of each model in predicting PMI status was then systematically compared.

Firstly, our study found that all models demonstrated good classification performance for predicting PMI status in CC in both the training and testing cohorts, with AUCs ranging from 0.842 to 1.000 in the training cohort and from 0.755 to 0.917 in the testing cohort. Several previous studies have reported the predictive performance of radiomics and deep learning models for PMI in CC, with AUCs ranging from 0.73 to 0.91 (11, 16), which is consistent with our findings. These results indicate that MRI-based radiomics can serve as a non-invasive biomarker for predicting PMI in CC, thereby helping to optimize treatment decisions.

Moreover, our study demonstrated that among the 15 radiomics models, the radiomics model based on T1C+ADC+DWI sequences achieved the best performance in predicting PMI in CC, with AUCs of 1.000 and 0.917 in the training and testing cohorts, respectively. This finding is consistent with Huang et al. (17), who showed that multiparametric MRI-based radiomics models outperform single-sequence models in predicting LVSI in CC. From a biological perspective, the superior performance of the T1C+ADC+DWI model may be attributed to the complementary information captured by the three sequences. T1C reflects tumor perfusion and neovascularization, which are closely related to tumor aggressiveness and stromal infiltration (18). ADC quantifies water diffusion within tissue and indirectly reflects tumor cellularity and microstructural integrity. DWI further enhances tissue contrast based on the diffusion of water molecules, aiding in the identification of tumor heterogeneity (19). This combination allows for a more comprehensive assessment of tumor biology, including microangiogenesis, blood supply, cellular density, and tissue structure, all of which are highly relevant to PMI. Interestingly, the model based on four sequences (T1C+T2WI+ADC+DWI) did not yield significantly better performance, suggesting that simply adding more sequences does not necessarily enhance predictive power. Instead, an optimal combination of informative and complementary sequences may be more effective for radiomics-based prediction of PMI in CC.

There are some limitations to this study. Firstly, this was a single-center study with a relatively small sample size. The model was developed and validated within the same cohort, which may limit the generalizability of the findings to broader clinical settings. Although the current results are encouraging, external validation using independent, multi-center datasets with larger and more diverse patient populations is necessary to confirm the robustness and clinical applicability of the model. Future research will focus on addressing this limitation. Secondly, this study was retrospective in nature, and selection bias was inevitable. Thirdly, although pathological type, degree of differentiation, LVSI (20), deep stromal invasion, and lymph node metastasis (21) are recognized pathological predictors of PMI in CC, these variables are only available through postoperative pathological examination and therefore were not included in the preoperative radiomics model. Fourthly, the ROIs in this study were manually delineated by experienced radiologists. Manual segmentation is time-consuming and subject to intra- and inter-observer variability, which may affect the reproducibility and clinical applicability of radiomics analysis. Although the Dice similarity coefficient was calculated to assess inter-observer agreement (mean Dice = 0.8566 ± 0.0442), intraclass correlation coefficient (ICC) analysis—which more directly evaluates the reproducibility of radiomic features—was not performed. In future work, we plan to incorporate ICC analysis to enhance the reliability of feature extraction and also to integrate automated or semi-automated segmentation tools to improve segmentation consistency and operational efficiency.

## 5 Conclusion

In conclusion, radiomics based on multiparametric MRI demonstrates excellent performance in predicting PMI in patients with early-stage CC. This noninvasive approach shows great promise for individualized preoperative assessment and may assist clinicians in optimizing surgical strategies while avoiding overtreatment or undertreatment. With further validation and integration into clinical workflows, radiomics models have the potential to contribute meaningfully to precision medicine and improve clinical decision-making in CC management.

## Data Availability

The original contributions presented in the study are included in the article/supplementary material. Further inquiries can be directed to the corresponding authors.
